# A High Accuracy AC+DC Current Transducer for Calibration

**DOI:** 10.3390/s22062214

**Published:** 2022-03-12

**Authors:** Xia Xiao, Hongtian Song, Hongbin Li

**Affiliations:** 1 School of Electrical and Electronic Engineering, Huazhong University of Science & Technology, Wuhan 430070, China; xiaoxiahust@163.com (X.X.); songhtsky@163.com (H.S.); 2 Southern Power Grid Research Institute Co., Ltd., Guangzhou 510663, China

**Keywords:** self-oscillating fluxgate, zero-flux, AC+DC current transducer, calibration, standard CT (current transformer)

## Abstract

Facing a lack of high accuracy current standards in the calibration of AC (Alternating Current) + DC (Direct Current) measurement devices that function to measure DC and AC simultaneously, a measurement method with high accuracy is proposed based on zero-flux self-oscillating fluxgate. An iron core and two windings are added onto the single-iron-core double-winding structure of the traditional self-oscillating fluxgate. The added iron core and its upper winding are used to weaken the influence of ripple on the sensor’s accuracy. The other one of the added windings is used for the feedback from the AC+DC magnetic potential, allowing the sensor to work in a zero-flux state and to measure AC+DC simultaneously. An AC+DC transducer prototype with an AC ranging from 0–500 A and DC 0–300 A is developed by selecting the core parameters and an optimized design of the circuit. The test results of the prototype show that the prototype can measure the AC and DC simultaneously, and the measurement accuracy reaches class 0.05 level in the nominal current range. This transducer can be used as a calibration standard of measurement devices for AC only, DC only, or AC and DC simultaneously. Compared with the AC+DC current transducer with the same accuracy level, the proposed transducer has fewer cores and simpler measuring circuit.

## 1. Introduction

DC in power grids is created due to induction from geomagnetic variations, DC power transmission, high-power electronic devices, and newly also by transformerless power inverters installed in solar power stations [1]. These currents, ranging in extreme cases up to hundreds of amperes, seriously affect the energy measurement and create a challenge in maintaining the DC-bias currents [2] at a certain level recommended by the grid codes [3]. This creates demand for the mixed AC+DC measurement, such as the DC-bias currents measurement [3] and AC measurement mixed with DC [4,5].

The standard CT used for the calibration of AC+DC measurement devices needs to have high AC and DC measurement accuracy under the condition of AC mixed with DC. The calibration of these AC+DC measurement devices require the input of AC mixed with DC. Due to the DC component, the accuracy of the standard CT based on the principle of electromagnetic induction will decline greatly and does not meet the requirements.

High-precision standard resistance is an ideal gauge of AC+DC, which is used as the calibration standard of anti-DC CT in Literature [6]. However, the high-precision standard resistance will consume power and heat up as the current increases.

Most of the reported high precision AC and DC measurement schemes are based on the magnetic modulation method. A precision DC/AC transformer is combined with a magnetic-modulation DC comparator and self-balancing AC comparator [7], which can measure AC and DC. The transformer consists of four iron cores and four windings. The electronic circuit includes an excitation oscillation circuit, peak detection circuit, amplifier circuit, and power amplification circuit. The errors at no DC component in the primary current are less than 20 ppm at currents between 1% and 100% of rated, and the errors, measured at DC between 10% and 100% of rated, are less than 100 ppm. There is no measurement data at AC mixed with DC. Meanwhile, the double core magnetic modulator has a false balance point, making its zero error larger.

Fluxgate magnetic modulation technology has the advantages of high resolution, wide measurement range, and high reliability. In the literature [8], a high-precision AC/DC proportional standard device based on the fluxgate and zero-flux principle was proposed. Two or four differential fluxgate sensors are symmetrically placed on the iron core to detect the residual magnetic potential caused by the imbalance between the primary and secondary currents. The accuracy of the prototype with a transformation ratio of 500:1 reaches level 0.01 at the power frequency AC 1–120% rated current and DC 1–120% rated current. Each fluxgate sensor requires external excitation. Additionally, to achieve the accuracy of level 0.01, the scheme requires four groups of fluxgate sensors to be symmetrically installed in the iron core to form a current sensor, which has high requirements for the processing technology of the iron core.

The magnetic modulator current sensing technology needs an external excitation source and a complex demodulation circuit. Meanwhile, the self-excited oscillation fluxgate sensor does not require an external excitation source, and its structure is simple. The operating principle of the open-loop self-oscillating fluxgate current sensor based on either the average current detection method [9,10] or the duty cycle method [11] has been studied. Compared with traditional fluxgate technology, the significant advantages of these sensors are their relatively simple modulation, demodulation circuits, and low cost.

However, due to the lower use of a magnetic core to compensate the modulation ripple induced in the primary and secondary windings due to the transformer effect, the obtained accuracy is only about 0.5% in the full scale of ±20 A [12,13].

A transducer combined with an improved self-oscillating fluxgate sensor with a magnetic integrator in a common feedback loop can measure DC up to ±600 A with a relative accuracy of 1.3 ppm in the full scale [14]. An iron core coil is added to the self-excited oscillation fluxgate sensor structure to weaken the conduction ripple in the iron core. The iron core coil and the self-excited oscillation fluxgate form a DC measurement channel. The third iron core detects the residual AC ripple in the iron core to further weaken the induced ripple in the iron core and form an AC measurement channel. In this paper, the researchers did not measure AC.

The main goal of this paper is to develop a standard current transducer for the calibration of AC+DC sensors with a simpler structure and sufficient accuracy. In this paper, a simpler AC+DC current transducer is proposed, which is composed of two iron cores and three windings. An iron core and two windings are added onto the single-iron-core double-winding structure of the traditional self-oscillating fluxgate. The added iron core and one of the windings are used to suppress the ripple in the iron cores. By matching core parameters and external circuit parameters, the ripple is suppressed to the greatest extent. In addition, through the LPF (low pass filter) and HPF (high pass filter) design in the electronic circuit, the self-excited oscillation fluxgate can measure AC and DC. The additional winding feeds back AC and DC to make the sensor cores work in a zero-flux state. In this way, the sensor only needs two iron cores and three windings to realize the high-precision simultaneous measurement of AC and DC.

The structure of this paper is as follows: the relevant existing works of AC/DC sensor, and fluxgate current sensor are reviewed in Section 1. Then, we introduce the single-core self-oscillating fluxgate current sensor principle in Section 2. In Section 3, the scheme for the AC+DC transducer is proposed in detail. In Section 4, the development of the AC+DC transducer is presented. We test the transducer to verify the accuracy and effectiveness of the scheme in Section 5. Finally, we summarize our work in Section 6.

## 2. Structure of Self-Oscillating Fluxgate Current Sensor

**Figure 1:**
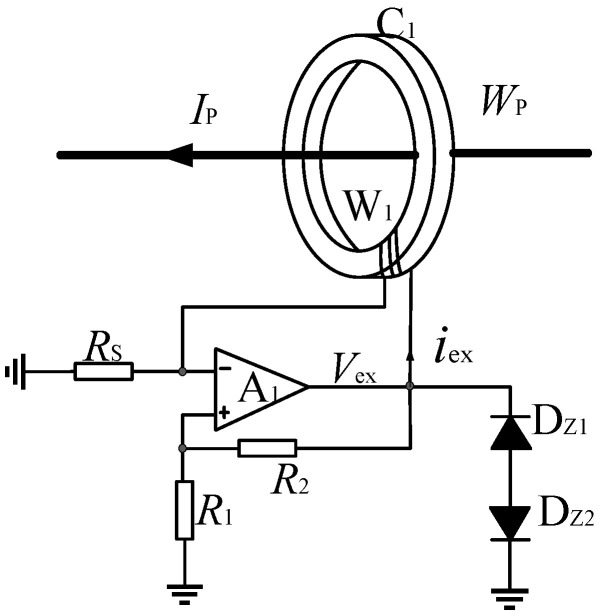
The self-oscillating fluxgate sensor circuit. $C_{1}$: iron core. $W_{1}$: excitation winding. $W_{p}$: primary winding. $I_{p}$: primary current. $A_{1}$: the comparator. $R_{1}$, $R_{2}$: the voltage dividing resistors. $R_{s}$: the sampling resistance. $D_{Z1}$, $D_{Z2}$: voltage limiting diode.

A schematic diagram of the open-loop self-oscillating fluxgate current sensor is shown in Figure 1. The operating principle of this circuit can be found in [9,10,11]. The excitation winding $W_{1}$ and the nonlinear core $C_{1}$ with high permeability and low magnetic saturation strength are equivalent to the nonlinear inductance L. The equivalent inductor L, the comparator $A_{1}$, the voltage dividing resistors $R_{1}$ and $R_{2}$, and the sampling resistance $R_{s}$ constitute a self-oscillating circuit.

When the self-oscillating fluxgate sensor operates, the excitation voltage $V_{ex}$ is a square wave signal with positive and negative symmetry. The iron core $C_{1}$ is in a state of alternating positive and negative saturation. Through piecewise linearization modeling on the excitation curve, it can be obtained that the period T of $V_{ex}$ meets

(1) $$T=\frac{4N_{1}B_{S}S}{V_{out}}$$

where $N_{1}$ is the number of turns of the excitation winding $W_{1}$. $B_{S}$ is the magnetic saturation strength of the core $C_{1}$. *S* is the cross-sectional area of core $C_{1}$. $V_{out}$ is the peak value of $V_{ex}$. Therefore, the excitation frequency of core $C_{1}$ can be calculated from the Formula (1).

According to the average current model [10], when the primary current is DC, during self-oscillating the average value $i_{av}$ of excitation current $i_{ex}$ meets

(2) $$i_{av}=-\frac{N_{P}I_{P}}{N_{1}}$$

It can be seen from Formula (2) that the average excitation current $i_{av}$ is proportional to the primary current $I_{P}$. Then the voltage generated by the exciting current $i_{av}$ on the sampling resistance $R_{S}$ can map the primary current $I_{P}$.

## 3. Principle of Zero-Flux Self-Oscillating AC+DC Transducer

### 3.1. Composition of AC+DC Transducer System

**Figure 2:**
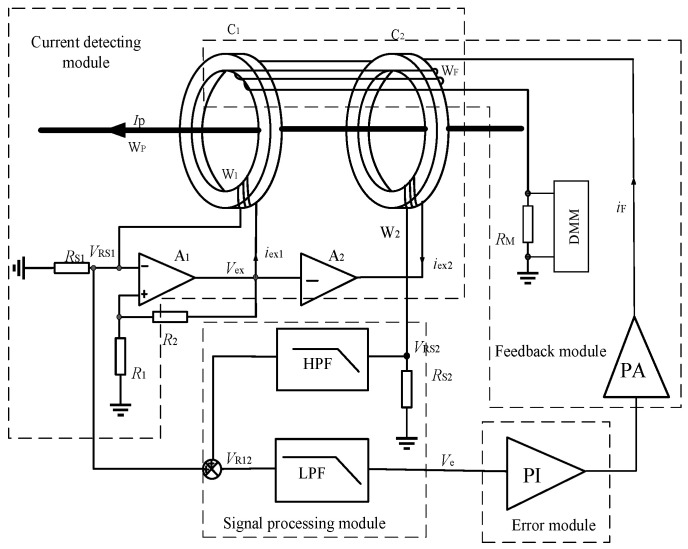
Composition of AC/C sensing system based on self-oscillating fluxgate principle. $C_{1}$, $C_{2}$: iron core. $W_{1},\;W_{2}$: excitation winding. $W_{F}$: feedback winding. $W_{p}$: primary winding. $I_{p}$: primary current. $A_{1}$, $A_{2}$: the comparator. $R_{1}$, $R_{2}$: the voltage dividing resistors. $R_{s1},\;R_{s2}$: the sampling resistance. $R_{M}$: the output sampling resistance. HPF: high pass filter. LPF: low pass filter. PI: proportional-integrator. PA: power amplifier.

In this paper, a zero-flux AC+DC sensing scheme based on the structure of self-oscillating fluxgate is proposed. See Figure 2 for its system composition. The self-oscillating flux gate serves as the zero-flux detector to detect AC+DC unbalanced magnetic potential, and the AC+DC feedback current flows through the feedback winding $W_{F}$ to make the cores in a zero-flux state. The ring core $C_{2}$, the exciting winding $W_{2}$, and the inverted amplifier $A_{2}$ reduces the electromagnetic induction noise, and the measuring accuracy is improved. The AC+DC sensing system is only of double-core three-winding configuration. Therefore, the iron core structure of the transducer is simplified and the overall cost is reduced.

In Figure 2, the sensing system includes the current detection module, signal processing module, error control module, and feedback module. In the current detection module, the ring iron cores $C_{1}$ and $C_{2}$ are nonlinear ferromagnetic materials of the same geometrical dimensions and magnetic parameters with high magnetic permeability, low coercive force, and high magnetic saturation induction strength. The excitation windings $W_{1}$ and $W_{2}$ with the number of turns $N_{1}$ and $N_{2}$ are evenly wound on the core $C_{1}$ and $C_{2},$ respectively. The comparator $A_{1}$, the winding $W_{1},$ and external resistances $R_{S1}$, $R_{1}$, and $R_{2}$ form a self-excited oscillation fluxgate. The square excitation voltage $V_{ex}$ is output from $A_{1}$. The amplifier $A_{2}$ is an opposite single-proportional amplifier. The exciting currents of cores $C_{1}$ and $C_{2}$ are of the same amplitude and opposite phase, and, therefore, the cores $C_{1}$ and $C_{2}$ operates in an opposite exciting state.

When the primary current flowing in the primary winding $W_{P}$ is $I_{P}$. The output voltage $V_{RS1}$ and $V_{RS2}$ on the resistor $R_{S1}$ and $R_{S2}$ is processed by the signal processing module, PI error module and PA circuit to generate feedback current in the feedback winding $I_{F}$. Finally, when the two iron cores are in zero flux state, the feedback current $I_{F}$ is proportional to the primary current. The magnetic potential balance equation for the toroidal cores $C_{1}$ and $C_{2}$ satisfies

(3) $$N_{P}I_{P}+N_{F}I_{F}=0$$

It follows from Formula (3) that when the sensing system reaches balance, the feedback current $I_{F}$ is proportional to the primary current $I_{P}$, and the transformation ratio is $N_{F}/N_{P}$. The voltage signal $V_{RM}$ output from the resistor $R_{M}$ and primary current $I_{P}$ shall satisfy the following requirements.

(4) $$I_{P}=-\frac{N_{F}}{N_{P}}I_{F}=-\frac{N_{F}}{N_{P}}\frac{V_{RM}}{R_{M}}$$

The sensitivity $S_{D1}$ can be derived from Equation (4):

(5) $$S_{D1}=-\frac{dV_{RM}}{dI_{P}}=-\frac{N_{P}R_{M}}{N_{F}}$$

Equation (5) shows that the sensitivity of the AC+DC transducer is proportional to the resistance of $R_{M}$ and inversely proportional to the number of turns $N_{F}$ of feedback winding.

### 3.2. Improvement of Transducer Performance by Zero-Flux

Due to the transformer effect, the square wave excitation flux of $C_{1}$ will induce the ripple current in two windings $W_{1}$ and $W_{2}$, which affects the accuracy of the transducer. To suppress the noise generated by the electromagnetic induction, the ring iron core $C_{2}$, excitation winding $W_{2}$, and the inverse amplifier $A_{2}$ are used to improve the performance of the transducer.

If there is no core $C_{2}$ with anti-excitation and the transducer is of a single core structure, then the magnetic potential equation of the core $C_{1}$ is

(6) $$N_{P}I_{P}+N_{F}I_{F}+N_{1}i_{ex}=0$$

From (6), it is known that excitation current $i_{ex}$ is still the main cause for the error of this transducer similar to traditional CTs.

Add a core $C_{2}$ with the same magnetic parameters and geometrical dimensions as $C_{1}$ and the excitation voltage $V_{ex}$ of core $C_{1}$ is inverted and directly used as the excitation voltage of core $C_{2}$. Here, the magnetic potential equations of the cores $C_{1}$ and $C_{2}$ are, respectively:

(7) $$N_{P}I_{P}+N_{F}I_{F}+N_{1}i_{ex1}=0$$

(8) $$N_{P}I_{P}+N_{F}I_{F}+N_{2}i_{ex2}=0$$

Add (7) and (8) together, i.e., the cores $C_{1}$ and $C_{2}$ are considered a whole, for the following:

(9) $$N_{P}I_{P}+N_{F}I_{F}=0$$

According to (9), the double-core self-oscillating fluxgate transducer can operate as a zero-flux state, thereby eliminating the impact on the measurement result of the single-core structure due to the electromagnetic induction, and achieving higher current detection accuracy of the AC+DC transducer.

### 3.3. Improvement of Transducer Performance by Demodulation Circuit Optimization

When the transducer measures the AC mixed with DC, the induced current on the excitation winding makes the excitation process of the iron core more complex and the nonlinear characteristics more obvious. A large number of high-frequency harmonics are generated in the excitation current. In this scheme, the high-frequency harmonic signals are effectively filtered out by the optimized demodulation circuit, and thus the measurement accuracy of the AC+DC transducer is improved.

When the primary current is the AC mixed with DC, the ring core $C_{1}$ is in the forward excitation state, and then the output voltage $V_{RS1}$ on the sampling resistor $R_{S1}$ consists of two parts: $V_{L1}$ and $V_{H1}$. $V_{L1}$ is a low-frequency signal, including a DC component $V_{dc}$ proportional to the primary DC and an AC component $V_{ac}$ proportional to the primary power frequency current. Meanwhile, a high harmonic current in the excitation signal also causes a high-frequency component $V_{H1}$. Since the magnetizing state of the toroidal core $C_{2}$ is precisely opposite to that of the core $C_{1}$, The output signal $V_{Rs2}$ on the sampling resistance $R_{s2}$ consists of $V_{L2}$ and $V_{H2}$ which are inverted with $V_{L1}$ and $V_{H1}$, respectively. High-pass filtering is performed on $V_{Rs2}$ and only the high-frequency component $V_{H2}$ is retained. Thanks to the symmetry of the sensor structure and parameters, in theory, the two signals $V_{H1}$ and $V_{H2}$ have equal amplitudes and inverse phases. Then add $V_{Rs1}$ and $V_{H2}$ to get

(10) $$V_{R12}=V_{Rs1}+V_{H2}=V_{L1}+V_{H1}+V_{H2}=V_{L1}$$

Equation (10) is an ideal output expression. However, the actual circuit cannot ensure consistency between $C_{1}$, $C_{2}$, and the additional circuit. It is impossible to eliminate the high-frequency component. It is necessary to filter the high-frequency component of the signal $V_{R12}$ further by low-pass filtering, to reduce the influence of electromagnetic induction. 

## 4. Development of AC+DC Transducer Prototype

### 4.1. Parameters of Cores

According to the requirements of a zero-flux self-oscillating flux transducer for the ferromagnetic parameters, the geometric and magnetic parameters of the cores $C_{1}$ and $C_{2}$ are summarized in Table 1.

### 4.2. Circuit Parameter

(1) Comparator

The performance parameters, such as power supply, load capacity, noise, and bandwidth of comparator $A_{1},$ are factors that affect the measurement accuracy of the transducer. A high-precision operation amplifier OP27G with a dual power supply is used. The supply voltage is limited to ±15 V, and the output current can reach 40 mA under 100 ohm load. Meanwhile, OP27G is characterized by a wide frequency bandwidth and low noise. The input offset current is less than 35 nA, and the unity gain-bandwidth product is 8 MHz. The phase inverter $A_{2}$ and comparator $A_{1}$ is of the same type.

(2) Excitation Frequency

From Formula (1), the relationship between the excitation frequency $f_{ex}$, excitation voltage, and core parameters is as follows:

(11) $$f_{ex}=\frac{V_{ex}}{4B_{S}N_{1}S_{C}}$$

The excitation frequency $f_{ex}$ should not be too high, since the vortex loss of magnetic material is proportional to the square of the excitation frequency $f_{ex}$. As the excitation frequency $f_{ex}$ increases, the vortex loss in the core will increase, and the overall power consumption of the AC+DC transducer will increase. From (11), when the excitation voltage $V_{ex}$ is constant, the higher the excitation frequency $f_{ex}$, the lower the number of turns $N_{1}$ of the excitation winding, and the higher the saturation current threshold of the core. Then, the core is difficult to enter the saturation area, which reduces the linearity of the transducer. 

While the excitation frequency $f_{ex}$ is too low, the number of turns $N_{1}$ of the excitation winding is too large, and the transducer’s sensitivity will be decreased. Therefore, it is necessary to focus on the linearity and sensitivity in the design of parameters. According to the condition of the excitation frequency limit $f_{ex}$ > 2f (f is the frequency of primary current), when the detection bandwidth of AC and DC sensor is 0–50 Hz, the excitation frequency of the self-oscillating fluxgate transducer shall be designed to be higher than 100 Hz. In the design, the number of turns $N_{1}$ of the excitation windings $W_{1}$ is 175, and the excitation square wave voltage is ±5 V. From (11) and the core parameters in Table 1, the excitation frequency of the AC+DC transducer is calculated to be 129 Hz, meeting the requirements for bandwidth detection.

(3) LPF and HPF

The function of HPF is to retain the high-frequency signal in excitation winding $W_{2}$, its cut-off frequency is set to 59 Hz. The output signal of the HPF is added to the output signal of the excitation winding $W_{1}$ to weaken the mutual inductance effect. To further filter out the high-frequency signal in the synthetic signal, a low-pass filter retaining only DC and power frequency signals is set, and its cut-off frequency is also set to 59 Hz. Therefore, the high-frequency ripple signal generated by the mutual inductance effect is greatly attenuated in the output signal $V_{e}$.

(4) Feedback circuit

**Figure 3:**
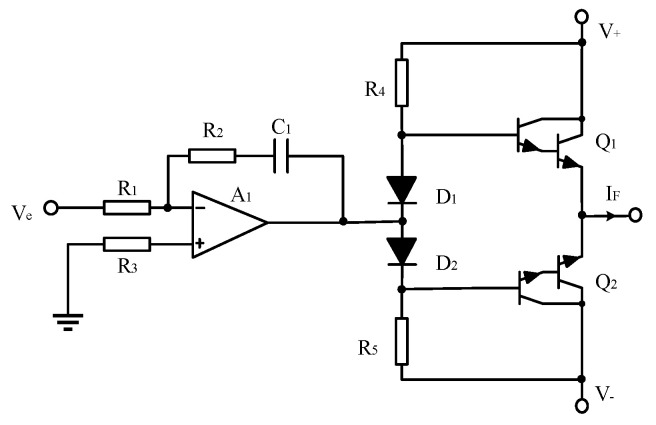
Schematic diagram of the power amplifier circuit.

To reduce the output ripple of the power amplifier circuit, a traditional AB-type power amplification circuit is applied in this design. The power devices $Q_{1}\;\mathrm{and}\;Q_{2}$ are TIP 110 and TIP 117, which have the same parameters, and are high-power Darlington tubes, with the maximum AC output as high as 2 A. The power amplifier circuit is shown in Figure 3.

### 4.3. Physical Transducer

**Figure 4:**
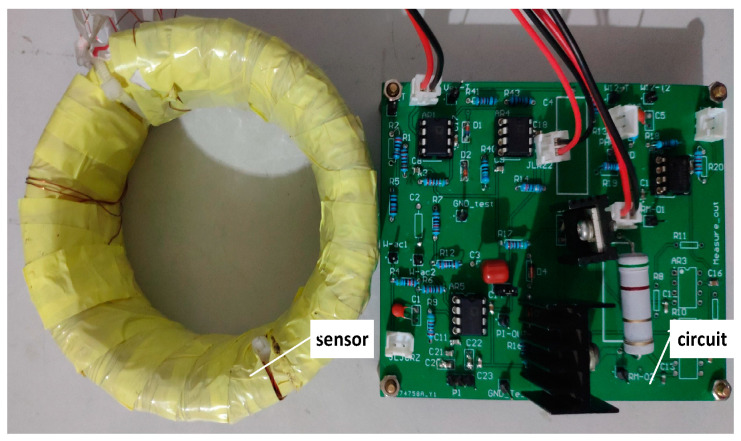
Prototype of zero-flux self-oscillating fluxgate AC+DC transducer.

Figure 4 shows the AC+DC transducer prototype based on the above principles and methods. Here are the main parameters of the given prototype. The transformation ratio: 1000:1; Rated AC: 500 A; Rated DC: 250 A; Sensitivity: 5 mV/A; and Bandwidth: 0–50 Hz.

## 5. Performance Test of AC+DC Transducer Prototype

**Figure 5:**
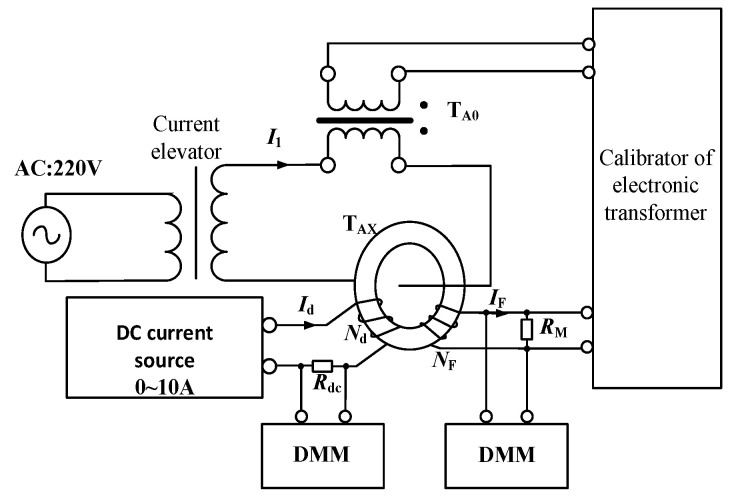
Schematic of error test based on proportional DC superposition method. $T_{A0}:$ Standard current transformer. $T_{AX}$: the tested high-precision AC+DC transducer prototype. $R_{M}$: the output sampling resistance. $N_{d}:$ winding turns of DC current.

**Figure 6:**
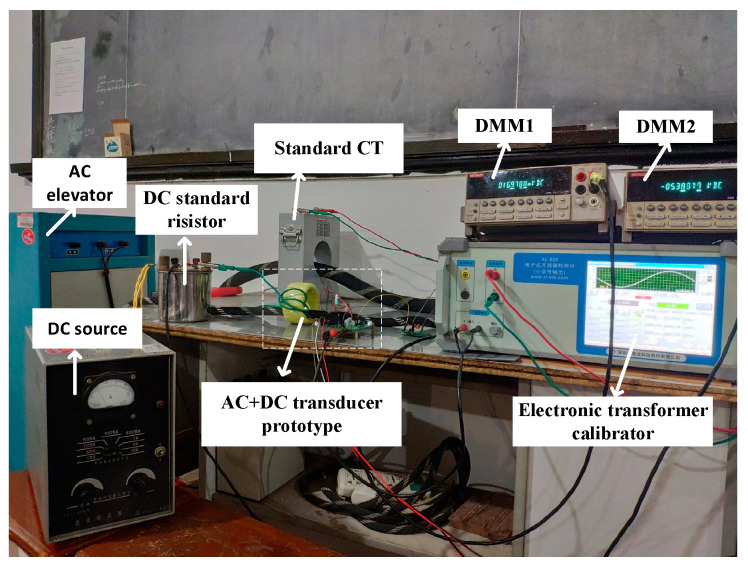
Photo of AC+DC transducer performance testing.

The AC+DC measuring performance of the prototype is tested based on the proportional DC superposition method. See Figure 5 for the test principle and Figure 6 for the wiring diagram. The test equipment and parameters are as follows.

(1) Current elevator. Capacity: 20 kVA; Maximum output current: 3 kA;
(2) Standard current transformer $T_{A0}$ Model: HL 28-5; Rated burden: 10 VA; Rated primary current: 500 A; Rated secondary current: 5 A; Accuracy class: 0.05;
(3) DC source. Model: YJ-10 A; Output current: 0;
(4) DC standard resistance $R_{DC}$ Resistance: 0.1 Ω; Capacity: 10 A at 20 °C; Temperature drift: 10 ppm; Accuracy class: 0.01;
(5) Calibration instrument of electronic transformers. Model: XL-809; Input range: 0–8 V; Accuracy class: 0.05;
(6) Six and a half digital multimeter DDM. Model: KEITHLEY 2000; DC voltage accuracy: 0.002%.

In Figure 5, $T_{AX}$ is the tested high-precision AC+DC transducer prototype. AC output by the elevator passes through $T_{AX}$ and standard current transformer $T_{A0}$. DC is produced by the DC source and loaded on $T_{AX}$ through equal ampere-turns. The output signal of $T_{AX}$ is obtained from the sampling resistor $R_{M}$ and input to the electronic transformer calibration instrument and DMM1 at the same time. The standard current transformer $T_{A0}$ converts the primary AC into low voltage and transmits it to the electronic transformer calibration instrument. The electronic transformer calibrator gives the AC verification results through synchronous sampling and calculation. The DC standard resistance transforms the primary DC into low voltage and transmits it to DMM2. The verification results of DC are calculated through the synchronous readings of the two DMMs. The ratio error and phase error are, respectively, calculated according to the standard definitions of the transformer, respectively, expressed in percentage and minute.

By the Proportional DC Superposition Method testing scheme, AC measuring performance, DC measuring performance, and AC/DC mixed current measuring performance of prototype are explored.

### 5.1. AC measuring Performance Testing

**Figure 7:**
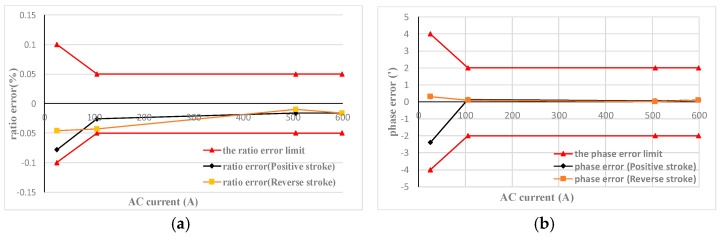
AC measuring performance test: (**a**) the ratio error for AC 0–600 A; and (**b**) the phase error for AC 0–600 A.

According to the Verification Code for Current Transformer for Measurement, the DC circuit was disconnected during testing, and the ratio error and phase error of the AC+DC transducer prototype with forward and backward strokes of 5%, 20%, 100%, and 120% of the rated current were, respectively, tested. The test results are shown in Figure 7a,b.

The red curve in Figure 7 is the limit curve of ratio error and phase error of the 0.05 class AC CT. It can be seen that the measured results of the prototype are within the error limits of the 0.05 class CT.

### 5.2. DC Measuring Performance Testing

**Figure 8:**
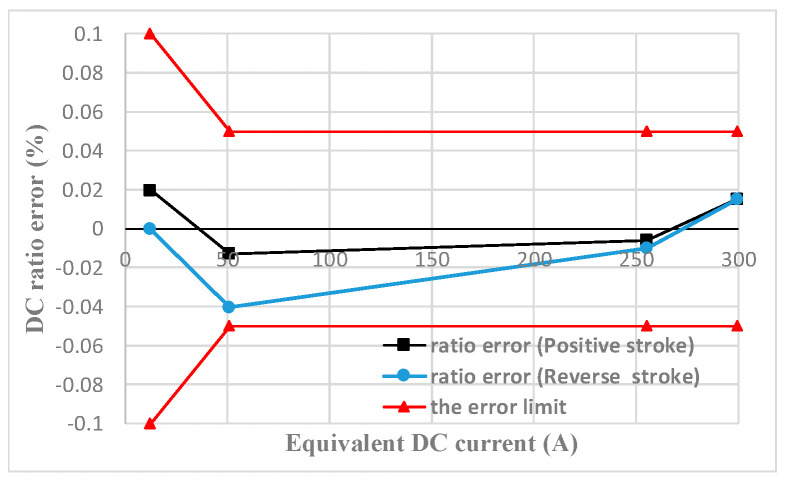
DC measuring performance test.

Referring to the Verification Code for Current Transformer for Measurement for DC characteristics testing, the equal ampere-turn method is used to extend the DC measuring range and the primary AC circuit is disconnected during testing. The test results within the range of 0–120% rated DC are shown in Figure 8.

The horizontal coordinate of Figure 8 is the equivalent primary DC and the vertical coordinate is the scale error. The red curve is the limit curve of the 0.05 class DC CT proportional error. It can be seen that the DC scale error of the transducer is within the error limit of 0.05 class CT in the DC range of 0–300 A.

### 5.3. AC+DC Measurement Performance Testing

Along with the Proportion DC Superposition method and the equal ampere-turn method, AC and DC are input simultaneously to explore the measurement performance when AC and DC are applied simultaneously, including the effects of DC component on AC measurement and AC component on DC measurement.

#### 5.3.1. Influence of DC Component on AC Measurement

**Figure 9:**
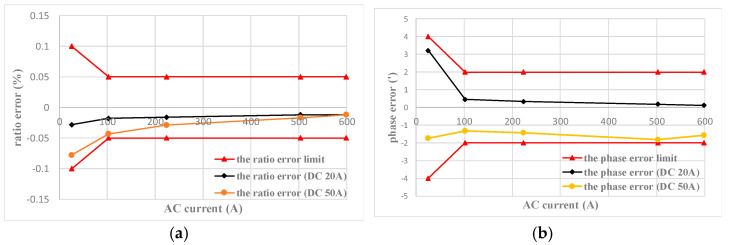
Influence of DC on AC measurement: (**a**) the ratio error for AC 0–600 A; and (**b**) the phase error for AC 0–600 A.

The ratio error and phase error of the AC+DC transducer prototype within the range of 0–600 A are tested when the DC component is fixed at 20 A and 50 A respectively. The test results are shown in Figure 9.

The red curve in Figure 9 is the limit curve of ratio error and phase error of the 0.05-class AC CT. It can be seen from Figure 9 that with a fixed DC of 20 A and 50 A, the ratio error and phase error of the AC+DC transducer does not change significantly, and still satisfies the AC error limit of the 0.05-class. It indicates that the DC component has no significant effect on the measurement of AC.

#### 5.3.2. Influence of AC on DC Measurement

**Figure 10:**
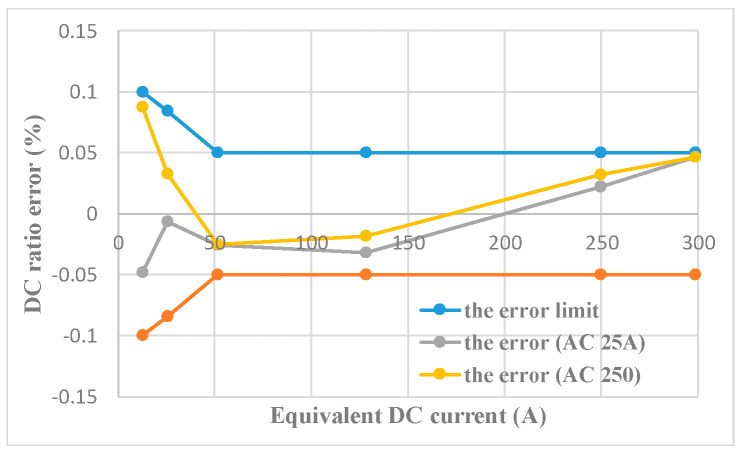
Influence of AC on DC measurement.

The DC scale error within the range of 0–300 A is tested when the AC is fixed at 25 A and 250 A, respectively. The test results are shown in Figure 10.

As shown in Figure 10, the red curve is the limit curve of the 0.05 class DC CT. It can be seen from Figure 10 that the DC measurement results still satisfy the 0.05 class DC error limit for an AC component of 25 A and 250 A. The magnitude of AC has no noticeable effect on the DC measurement error.

## 6. Conclusions

The measurement accuracy of the standard CT used for AC CT calibration cannot meet the calibration when AC and DC currents are mixed. It requires a high accuracy standard current transducer that can measure AC and DC simultaneously. An AC+DC transducer based on zero-flux self-oscillating fluxgate is proposed in this paper. Some parameters of the transducer are: Rated AC: 500 A; Rated DC: 250 A; Bandwidth: 0–50 Hz. The ratio error and phase error of the transducer prototype are within the error limit of class 0.05 CT at the range of 5–120% of the rated current When only DC or AC. The errors of the transducer are also tested at AC mixed with DC and DC mixed with AC. The ratio error and phase error of the prototype at the AC range of 0–600 A are within the error limit of class 0.05 CT when the DC component is fixed at 20 A and 50 A, respectively. The DC scale error at the range of 0–300 A is within the error limit of class 0.05 CT when the AC is fixed at 25 A and 250 A, respectively. The transducer can calibrate AC CTs, DC CTs, and anti-DC CTs with an accuracy level not high than 0.2.

The AC+DC transducer proposed in this paper has a simpler structure and only needs double cores and three windings. However, at present, the sensor has high accuracy only under the power frequency AC mixed with DC; that is, its bandwidth is limited. The research team is trying to expand the bandwidth of the sensor by changing the excitation frequency of the self-excited oscillation circuit and the matching of circuit parameters to make it more widely used, such as the calibration under half-wave current.

## Figures and Tables

**Table 1:** Geometrical and magnetic parameters of cores.

Parameters	Actual Meaning	Value	Unit
*D*_1_	Outer diameter of core	85	mm
*D*_2_	Inner diameter of core	75	mm
$S_{C}$	Core cross-section area	0.5	${\mathrm{cm}}^{2}$
$L_{e}$	Effective magnetic circuit length	25.12	cm
$B_{S}$	Saturated magnetic flux density	1.1	T
$B_{r}$	Residual magnetic flux density	0.66	T
$H_{c}$	Coercive force	0.6	A/m
$\mu_{i}$	Initial relative magnetic permeability	200,000	-
$\mu_{M}$	Maximum relative magnetic permeability	600,000	-

## Data Availability

The data used to support the findings of this study are available from the corresponding author, [X.X.] and [H.S.], upon request.
